# From text to traits: exploring the role of large language models in plant breeding

**DOI:** 10.3389/fpls.2025.1583344

**Published:** 2025-05-14

**Authors:** Mohsen Yoosefzadeh-Najafabadi

**Affiliations:** Department of Plant Agriculture, University of Guelph, Guelph, ON, Canada

**Keywords:** artificial intelligence, computational biology, knowledge graph, plant breeding, plant omics

## Abstract

Modern plant breeders regularly deal with the intricate patterns within biological data in order to better understand the biological background behind a trait of interest and speed up the breeding process. Recently, Large Language Models (LLMs) have gained widespread adoption in everyday contexts, showcasing remarkable capabilities in understanding and generating human-like text. By harnessing the capabilities of LLMs, foundational models can be repurposed to uncover intricate patterns within biological data, leading to the development of robust and flexible predictive tools that provide valuable insights into complex plant breeding systems. Despite the significant progress made in utilizing LLMs in various scientific domains, their adoption within plant breeding remains unexplored, presenting a significant opportunity for innovation. This review paper explores how LLMs, initially designed for natural language tasks, can be adapted to address specific challenges in plant breeding, such as identifying novel genetic interactions, predicting performance of a trait of interest, and well-integrating diverse datasets such as multi-omics, phenotypic, and environmental sources. Compared to conventional breeding methods, LLMs offer the potential to enhance the discovery of genetic relationships, improve trait prediction accuracy, and facilitate informed decision-making. This review aims to bridge this gap by highlighting current advancements, challenges, and future directions for integrating LLMs into plant breeding, ultimately contributing to sustainable agriculture and improved global food security.

## Introduction

Plant breeding is the process of developing new cultivars that begins with selecting potential parental lines with desirable traits and making crosses to create a breeding population, followed by implementing various selection strategies to identify superior lines (Yoosefzadeh-Najafabadi and Rajcan, 2023). At the end of the breeding cycle, superior lines will be selected based on different traits of interests including disease resistance, increased yield, improved flavor, and better adaptability to various environmental conditions (Yoosefzadeh-Najafabadi and Rajcan, 2023). However, most of the traits are complex in nature, controlling by several genes with major and minor effect, environment, management, and their interactions with other omics such as transcriptomics, metabolomics, proteomics, etc (Haq et al., 2023). Therefore, plant breeders leverage various tools and disciplines to enhance the pace of crop improvement and make their selection more accurate.

One of the most important tools that breeders have been utilizing extensively for decades is the use of mathematical and statistical approaches. These approaches act as a guiding compass, empowering breeders with two powerful wings to navigate through the vast landscape of datasets, skillfully evaluate lines and predict their performance under various climate and management conditions. At the beginning, analysis of variance (St and Wold, 1989) and *post hoc* comparison test (Williams and Abdi, 2022) were become incredibly popular within the plant breeding community, with the use of α ≤ 0.05 (commonly) as the threshold, pioneered by Fisher (Fisher, 1936). It is worth noting that Fisher unintentionally established this threshold to distinguish between significant and non-significant results (Stigler, 2008).

Gradually, breeders have utilized more approaches as they broaden their research into comparing different environments, the genetic and environment interactions, understanding the relationships between important agronomic traits and evaluating varieties based on multiple traits. Methods such as Principal Component Analysis (Abdi and Williams, 2010), Multidimensional Scaling (Douglas Carroll and Arabie, 1998), factor analysis (Lawley and Maxwell, 1962), stability analysis (Lin et al., 1986), and the Additive Main Effects and Multiplicative Interaction model (Moreno-González et al., 2003) have played a significant role in helping breeders to understand the fact that successful breeding is not merely a matter of trial and error in the field or probabilities of success by increasing the number of crosses, but rather a nuanced process that involves significant understanding of intricate relationships within/among traits and the art of superior selection.

In order to facilitate their understating, breeders have begun incorporating multi-omics into their research, but identifying interactions continues to present challenges (Yoosefzadeh Najafabadi et al., 2024). Many existing tools rely on comparing P-values derived from two variables at a time, which is not well-equipped to handle multiple variables simultaneously (Yoosefzadeh Najafabadi et al., 2023b). Additionally, adhering strictly to the traditional significance threshold of α ≤ 0.05 may result in overlooking valuable variables throughout the analysis, further complicating the situation. Furthermore, as data collection technologies advance with high-throughput omics approaches, vast amounts of data are being generated at an unprecedented rate; the concept of big data is beginning to make waves in the field of breeding (Hina et al., 2024). This abundance of data poses challenges for current methods, particularly due to the “small n, large p” problem and the diverse nature of the data points, making accurate analysis and interpretation an intimidating task.

In recent decades, plant breeders started to incorporate more sophisticated approaches such as machine/deep learning algorithms in order to overcome the shortcomings of conventional algorithms. One of the key advantages of machine learning (ML) is its ability to handle big data generated by high-throughput omics approaches, which is crucial in the field of breeding where vast amounts of data are being produced rapidly (Yoosefzadeh Najafabadi et al., 2023b). Gradually, breeders have recognized the potential benefits of combining multiple ML algorithms into ensemble models to further enhance their analysis of multi-omics data. Ensemble algorithms work by aggregating the predictions of multiple base models to generate a final prediction that often outperforms any individual model. By leveraging ensemble techniques such as random forests, gradient boosting, or stacking, breeders can harness the strengths of different algorithms and mitigate their weaknesses, leading to more robust and accurate predictions (Montesinos-López et al., 2024). Deep learning (DL), a subset of machine learning, offers even more advanced capabilities for breeders looking to analyze complex multi-omics data (Farooq et al., 2024). DL algorithms, particularly neural networks with multiple layers, excel at automatically learning intricate patterns and representations from large and diverse datasets (Yoosefzadeh Najafabadi et al., 2023c; Farooq et al., 2024). This is especially beneficial in breeding research where the data often exhibits non-linear relationships and interactions that are difficult to capture using traditional methods. Furthermore, DL algorithms can adapt and improve their performance over time as they are exposed to more data, making them well-suited for handling the evolving nature of multi-omics data in breeding research (Farooq et al., 2024). There is no doubt that classical ML and DL algorithms can process large datasets and detect patterns; however, they often require extensive feature engineering and may struggle with integrating diverse data types simultaneously, such as genomic, phenotypic, and environmental data. These models are typically domain-specific, focusing on individual aspects of the data rather than offering a holistic view. Consequently, they might overlook subtle genetic interactions and fail to capture non-linear relationships comprehensively (Yoosefzadeh Najafabadi et al., 2023b).

As breeders strive to decode complex biological aspects behind different traits of interests and refine crop improvement strategies, Large Language Models (LLMs) offer a groundbreaking approach (Kuska et al., 2024; Lam et al., 2024). LLMs, with their advanced capabilities in understanding and generating human-like text, offer great potential in synthesizing vast and diverse datasets. Their application in plant breeding can revolutionize how breeders’ access, interpret, and utilize information from high-throughput omics technologies, phenotypic, and environmental sources (Kuska et al., 2024). Unlike traditional methods, LLMs can manage vast and heterogeneous datasets by leveraging their ability to uncover intricate relationships without domain-specific feature engineering. By analyzing vast amounts of data, LLMs can extract insights, identify patterns, and even generate novel hypotheses that can steer breeding programs towards more informed and efficient decision-making (Lam et al., 2024; Pan et al., 2024). This, in turn, can streamline the breeding process, reduce time and resources spent on trial and error, and help in the development of more resilient and productive crop varieties.

The primary goal of this paper is to elucidate the transformative potential of LLMs in the field of plant breeding. By integrating DL and sophisticated AI techniques, the paper aims to demonstrate how LLMs can handling vast and complex datasets to provide a comprehensive understanding of biological aspects behind complex traits. The paper seeks to highlight the specific applications of LLMs in plant breeding, from improving the accuracy of predictions to enabling the discovery of novel genetic interactions. Additionally, it aims to provide a comprehensive guide on the implementation of LLMs, showcasing its potential in plant breeding area. Ultimately, this paper aspires to pave the way for a more informed and data-driven approach to plant breeding, fostering innovation and efficiency in the development of superior crop varieties.

## Evolution of plant breeding: from early practices to advanced computational techniques

**Figure 1:**
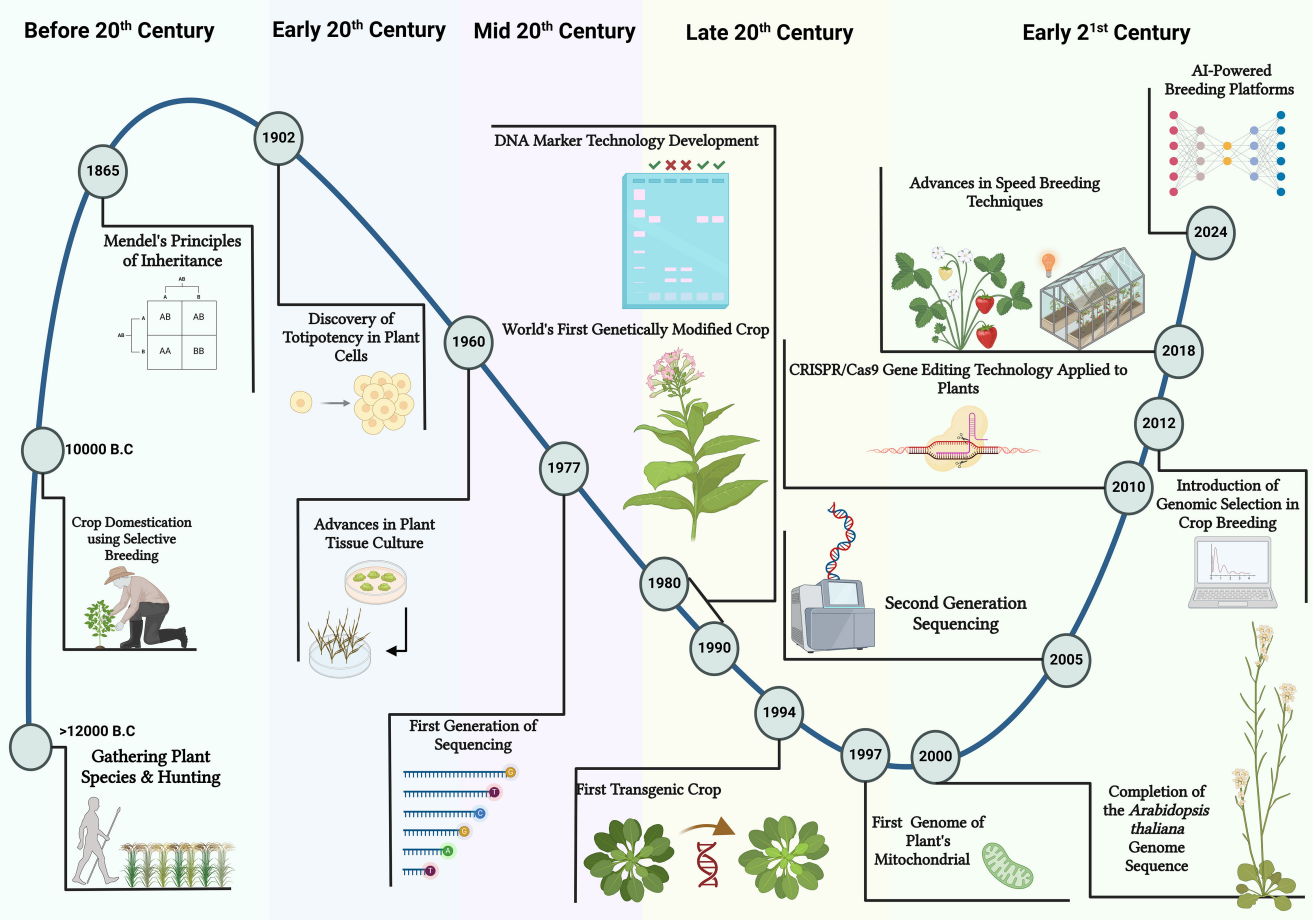
An illustrated timeline presents a historical perspective on plant breeding techniques. It started with the Crop Domestication phase, when selective breeding began around 10,000 BC. The next era, Conventional Breeding, involved the use of systematic selection and hybridization to improve desirable traits. In the 1980s, Molecular Breeding ad marker assisted selection was introduced, advancing genetic mapping and molecular markers to enable DNA-level trait selection. Predictive Breeding utilized in 2012, integrating genomic data with advanced analytics for more efficient and accurate selection. As plant breeding moves forward, AI-Powered breeding platforms are being developed, representing the next frontier in plant breeding. This illustration was created using BioRender.com.

Plant breeding is a dynamic field that has undergone significant evolution over the years (**Figure 1**). The origins of plant breeding date back centuries, coinciding with the advent of agriculture itself (Lee et al., 2015). Early farmers intuitively selected plants with desirable traits for cultivation, laying the groundwork for what would become a sophisticated scientific discipline. The formalization of plant breeding as a scientific endeavor began in the 19^th^ century with the work of Gregor Mendel, whose experiments with pea plants established the principles of heredity (Yoosefzadeh Najafabadi et al., 2023c). Mendel’s laws provided a foundational framework for understanding how traits are inherited, enabling breeders to predict the outcomes of their breeding activities (Yoosefzadeh Najafabadi et al., 2023c). Over time, this knowledge facilitated the development of more structured and systematic approaches to plant breeding (**Figure 1**).

The 20^th^ century witnessed remarkable advancements in plant breeding driven by the adoption of genetics and biotechnology (Kim et al., 2020). The Green Revolution, marked by the introduction of high-yielding varieties and the use of chemical fertilizers and pesticides, significantly boosted agricultural productivity worldwide and helped avert widespread famine (Conway and Barbie, 1988). However, this period also highlighted the importance of addressing issues such as genetic diversity and environmental sustainability. Conventional breeding methods, such as mass selection, backcrossing, and hybridization, became staples of the plant breeding process, allowing breeders to develop varieties with improved traits like yield, disease resistance, and stress tolerance (Singh et al., 2021). Despite these successes, the intricate nature of traits governed by complex genetic architectures and environmental interactions posed ongoing challenges.

The transition from conventional plant breeding to modern breeding techniques reflects the broader shift towards data-driven and precision agriculture (Farooq et al., 2024). As traditional methods reached their limits in addressing complex challenges such as climate change, food security, and resource sustainability, breeders began to explore innovative approaches that integrate technological advancements with classical breeding principles. One of the most transformative changes has been the integration of multi-omics technologies, including genomics, transcriptomics, proteomics, and metabolomics, which allow for a holistic examination of the biological processes underlying trait expression (Hina et al., 2024). This integration provides a multidimensional understanding of how genes, proteins, and metabolites interact, enabling breeders to gain insights into trait variation and stress responses.

The integration of advanced computational techniques is a defining feature of modern plant breeding (**Figure 1**). These tools have transformed the analysis of complex datasets, enabling breeders to identify hidden patterns and relationships that were once difficult to detect (Farooq et al., 2024). However, the sheer volume and heterogeneity of multi-omics data have outpaced the capabilities of traditional computational methods, which often require structured inputs and struggle to synthesize unstructured sources such as scientific literature or field notes. This is where LLMs emerge as a recent advancement in the evolution of plant breeding, building on the foundation laid by earlier computational techniques while addressing their limitations. Rooted in transformer architectures developed for natural language processing, LLMs excel at processing sequential and textual data, ranging from sequences to research publications, without the need for extensive preprocessing or domain-specific feature engineering (Lam et al., 2024). This capability marks a significant leap beyond the trial-and-error approaches of early breeding and the data-limited precision of mid-20th-century methods, positioning LLMs as a cornerstone of modern, data-driven breeding programs. However, to effectively implement recent algorithm advancements into the breeding program, breeders need to utilize a wide array of packages and libraries available in various programming languages, including R, Python, and Bash (Yoosefzadeh Najafabadi et al., 2023a). This raises the important question of how coding and computer languages are empowering plant breeders to address the big data challenges arising from the use of multi-omics in their breeding programs.

### How codes are helpful in plant breeding?

The rapid integration of new technologies in breeding programs has led to a significant increase in the volume, variety, and accuracy of data points, combined with the nature of data collection, thereby presenting big data challenges (Yoosefzadeh-Najafabadi et al., 2024). Historically, concerns over the storage, analysis, and interpretation of multi-omics datasets within constrained timeframes posed significant challenges to their adoption in advancing breeding programs. However, these challenges are gradually being mitigated through the availability of diverse software packages and platforms developed in various programming languages, such as R, Python, and Bash (Kim et al., 2020). Additionally, the implementation of AI components, including ML, DL, reinforcement learning (RL), and transfer learning (TL), has fostered effective collaboration between plant and computer scientists, facilitating the extraction of valuable information from multi-omics datasets (Kim et al., 2020; Farooq et al., 2024).

Coding plays an important role not only in data analysis but also in streamlining data integration processes. Modern data integration techniques have proven effective in evaluating complex traits, such as soybean yield. For example, Yoosefzadeh-Najafabadi et al. (2021) introduced a hyperspectral genome-wide association study (HypWAS) using a hierarchical data integration strategy to assess the predictive power of hyperspectral reflectance bands for soybean seed yield. This comprehensive analysis was executed in R, utilizing various packages that facilitate these complex computations. In a similar area, ML and DL algorithms have been applied in plant breeding programs to detect biotic and abiotic stresses in crops, such as stripe rust in wheat (Walsh et al., 2024), iron deficiency chlorosis in soybeans (Xu et al., 2021), and powdery mildew in vegetables (Mahmood ur Rehman et al., 2024). These studies utilized different programming languages and extensive coding to optimize algorithm parameters, visualize data, and interpret results. Therefore, the use of coding and computational tools in plant breeding is vital for future advances, unlocking deeper insights and fostering innovations that improve crop resilience and productivity. In terms of coding for LLMs, an LLM coded in Python could be fine-tuned on a corpus of plant breeding publications to extract insights about genetic markers linked to drought tolerance, then integrate these with hyperspectral data from HypWAS to refine yield predictions under water-limited conditions. Moreover, LLMs can streamline bioinformatics workflows by assisting in coding tasks, such as generating R scripts to analyze multi-omics data or debugging Python code for genomic annotations, reducing the technical burden on plant breeders (Zhao et al., 2023).

### Does algorithm help plant breeders?

Algorithms are central to the computational toolkit in plant breeding, where they serve numerous critical functions. An algorithm is essentially a step-by-step procedure or formula for solving a problem, and in the context of plant breeding, they are used to process and analyze large volumes of data with speed and accuracy that would be unattainable through manual methods (Yang et al., 2021). From simple statistical calculations to complex ML models, algorithms enable breeders to examine genetic diversity, estimate breeding values, and identify omics regions associated with desirable traits (Yang et al., 2021). For instance, algorithms used in predictive modeling can help estimate the potential yield or disease resistance of future plant generations, thereby improving the selection and breeding of superior cultivars (Cooper et al., 2014). The utilization of ensemble methods such as random forests and gradient boosting further enhances predictive accuracy by combining the strengths of multiple algorithms (Zhang et al., 2022).

Furthermore, algorithms facilitate the exploration of genetic relationships, such as epistatic interactions and genotype-environment interactions, which are important for understanding the full complexity of trait expression (Dwivedi et al., 2024). Advanced algorithms in DL, particularly neural networks, go a step further by automatically learning representations of data, optimizing breeders’ abilities to forecast breeding outcomes and design efficient breeding experiments (Montesinos-López et al., 2021). Algorithms, therefore, provide plant breeders with a powerful means to harness the potential of big data, enabling precise and informed intervention in the breeding pipeline (Mansoor et al., 2024).

The advent of LLMs elevates the role of algorithms in plant breeding by introducing a versatile, data-agnostic approach that exceeds the limitations of traditional methods. For example, an LLM could analyze genomic sequences and environmental data alongside unstructured field trial notes to predict epistatic effects on yield with greater nuance than random forests, which rely on pre-engineered features. Similarly, LLMs can synthesize multi-omics data to predict genotype-environment interactions under future climate scenarios, providing breeders with actionable crossing recommendations. Unlike domain-specific DL models, LLMs offer adaptability through fine-tuning or zero-shot learning, allowing breeders to repurpose them for diverse tasks, such as annotating regulatory regions in wheat genomes or generating hypotheses about stress tolerance genes by processing thousands of research articles (Kuska et al., 2024). This flexibility reduces the need for multiple specialized algorithms, streamlining workflows and enhancing precision. By integrating LLMs into breeding pipelines, algorithms evolve from mere data processors to intelligent partners, capable of uncovering novel insights and accelerating the development of superior cultivars with unprecedented efficiency.

## Leverage the best of existing datasets, findings, and innovations

As plant breeders leverage advanced algorithms and multi-omics datasets to unravel the complexities of complex traits, they are generating a wealth of insightful results that drive the field forward. Furthermore, the valuable datasets derived from multi-omics explorations are frequently archived in platforms such as NCBI and other repositories, ensuring broader accessibility and preservation for future research endeavors (Misra et al., 2019; Binokay et al., 2025).

**Figure 2:**
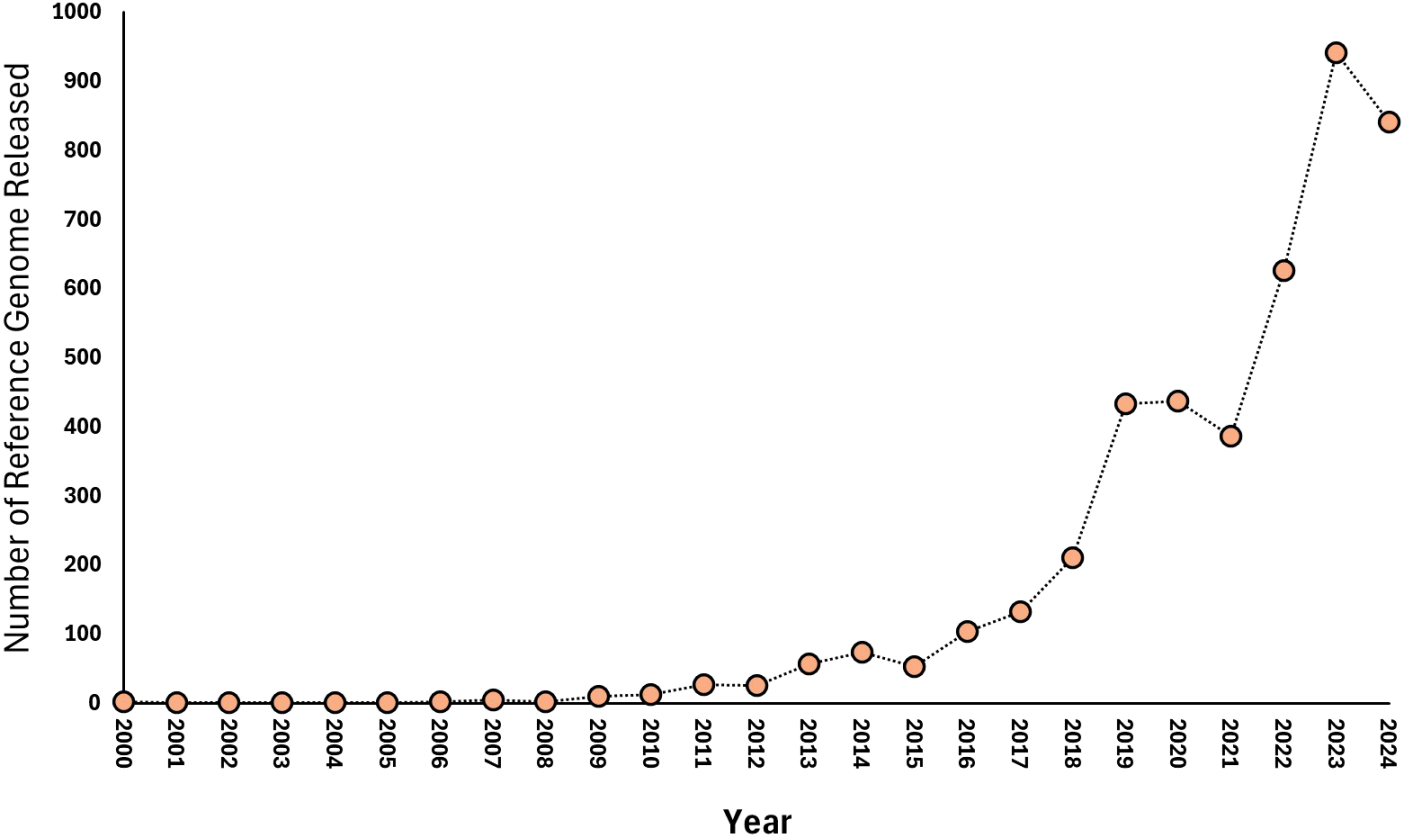
**A** figure showcasing the release of plant reference genome data in the NCBI database ranging from 2000 to 2024.

The trend in the release of plant-related reference genome data from 2000 to 2024 (NCBI, 2024) reveals a remarkable increase in the availability of sequencing data pertinent to plant breeding and genomics (**Figure 2**). The gradual rise in reference genome releases from only a couple of datasets in the early 2000s to a peak of 942 in 2023 (NCBI, 2024) underscores the significant advancements in sequencing technologies and their increasing adoption within the field of plant research. In the initial years, specifically from 2000 to 2009, the number of plant-related reference genome releases was minimal, with only a total of 10 datasets published by 2009 (NCBI, 2024). This limited output can be attributed to several factors, including the relatively high cost of sequencing, the early stages of technology development, and the lack of widespread application in plant breeding research. During this time, most studies focused on foundational genomic research rather than large-scale data generation. A significant turning point occurred in the early 2010s, particularly from 2010 to 2014, when the number of plant-related reference genome releases began to grow exponentially (NCBI, 2024). For instance, releases increased from 13 in 2010 to 74 in 2014 (**Figure 2**). This growth can be attributed to various factors, including advancements in sequencing technology, increased focus on genomic research in plants, and collaborative initiatives and consortia. The exponential rise in reference genome releases from 2018 onwards, with records peaking at 942 in 2023 (NCBI, 2024), reflects the culmination of these trends. Factors contributing to this increase include emerging applications in precision plant breeding, regulatory and funding support, and open data initiatives. The sustained high volume of reference genome releases in recent years highlights the growing importance of genomic resources for plant breeding (Yoosefzadeh-Najafabadi et al., 2024). These datasets provide valuable insights into genetic diversity, trait associations, and genomic architectures, enabling breeders to make more informed decisions and enhance breeding efficiency (Yoosefzadeh-Najafabadi et al., 2024). As the volume of available data continues to grow, there is an increasing need for advanced bioinformatics tools and analytical frameworks to effectively utilize these resources in plant breeding strategies.

Despite the wealth of data and insights being generated, effective integration into breeding programs remains a key challenge. This is where LLMs become crucial, by offering a transformative approach to navigate and synthesize the vast repositories of knowledge scattered across publications and online databases (Lam et al., 2024). LLMs can serve as intelligent intermediaries, providing strategic access to existing data and facilitating its incorporation into individual breeding initiatives. To expand this potential, LLMs can leverage the growing datasets in novel ways not yet fully explored in plant breeding. For instance, an LLM could be trained on the 942 plant reference genomes from 2023 (NCBI, 2024) alongside real-time satellite imagery data to model how genetic variations influence canopy development across diverse agroecosystems, offering breeders spatially explicit insights for selecting climate-adaptive cultivars. LLMs also could integrate multi-omics datasets with emerging single-cell sequencing atlases, such as those mapping root responses to nutrient deficiencies, to predict how cellular-level gene expression translates to whole-plant phenotypes, a granularity beyond the reach of conventional bioinformatics pipelines. By constructing dynamic knowledge graphs that evolve with new data inputs, LLMs can track temporal trends in trait evolution, such as shifts in disease resistance profiles over decades, enabling breeders to predict pathogen pressures and prioritize resistant germplasm. These innovative applications demonstrate how LLMs can transform static datasets into living, predictive tools, bridging the gap between data generation and application to drive rapid, impactful advancements in crop development.

## The story of language models, the definition and basic information

Language models (LMs) consist of advanced algorithms or neural networks that are trained extensively on large text datasets to learn and identify statistical relationships and patterns in natural language (Lam et al., 2024). LMs have a long history of use in biological applications, functioning as word n-grams, convolutional neural networks (CNNs), long short-term memory (LSTM) networks, and transformers (Anderson et al., 2021; Lam et al., 2024).

Word n-grams, an important type of LM, are sequences of n consecutive words in a given text, where ‘n’ is a positive integer (Anderson et al., 2021). For example, the term “Xyloglucan endo-transglycosylase” forms a 2-bigram. Word n-grams are typically used in text mining within scientific publications and for identifying regulatory elements in DNA sequences (where n-grams and k-mers are often used interchangeably), as well as for interpreting protein-protein interactions (Pan et al., 2022). However, n-grams have a major drawback as they cannot account for the order of words, which means they fail to capture the complex context that exists between different n-grams or k-mers. Therefore, they cannot fully capture the biological aspects of a trait of interest, such as the order of genes, phenotypes, or the best sequence for making crosses (in plant breeding area), which is a challenging task using this approach. CNNs, another type of LM, use convolutions, essentially filters, to analyze images or sequences of characters (Zrimec et al., 2020; Gao et al., 2022). These filters are employed to detect specific features or information within the input data. In plant biology, CNNs have been crucial for identifying regulatory enhancers in DNA and have been used in studying protein ubiquitination (Gao et al., 2022). However, like n-grams, CNNs have limitations due to the fixed size of their filters, making them better suited to capturing local patterns rather than understanding long-range dependencies or complex sentence structures (Zrimec et al., 2020).

Despite these limitations, CNNs have performed well in the fields of genomics and phenomics. They have been widely used to predict gene expression levels based on sequence data (Zrimec et al., 2020). By incorporating techniques such as dilation and scanning field approaches, CNNs have outperformed other neural network models (Erfanian et al., 2023). They have shown a strong ability to identify significant motifs in input sequences and have been extensively used in genomics and transcriptomics analyses because of their unique strengths (Washburn et al., 2019; Erfanian et al., 2023). Additionally, CNNs have been successfully used to predict the sequence specificities of DNA and RNA-binding proteins (Washburn et al., 2019). In phenomics, CNNs are used to analyze plant images to assess traits like leaf size, shape, number, and health, aiding in understanding plant growth and development under various conditions (Mansoor et al., 2024). For example, CNNs can automatically classify different plant species or detect various disease symptoms from leaf images, enhancing breeding programs and crop management (Ngugi et al., 2021; Iftikhar et al., 2024).

LSTM models are a specialized type of Recurrent Neural Network (RNN) that excel in processing sequential data, such as text and multi-omics sequences (Lam et al., 2024). These models are skilled at capturing long-range dependencies in data through the use of both long and short-term memory constructs (Gandhewar et al., 2025). LSTMs are applied in biology for tasks such as genome annotation and genotype classification (Taghavi Namin et al., 2018; Gandhewar et al., 2025). However, a limitation of LSTMs, as well as other RNNs, is their tendency to lose track of information from the beginning of a sequence when dealing with longer texts (Amiri et al., 2024). This problem arises due to the vanishing gradient issue, where the model’s memory fades as information is compressed over time. Additionally, LSTMs are prone to the exploding gradient problem, which can cause instability and training difficulties for certain datasets (Turkoglu et al., 2022). The sequential nature of LSTM processing also hinders its training efficiency, as it cannot utilize parallel computation, resulting in slower and more resource-intensive training cycles (Turkoglu et al., 2022).

In contrast, Transformer models, introduced in 2017 to improve machine translation (Vaswani, 2017), have since been applied to a wide range of genomic challenges (Avsec et al., 2021; Ji et al., 2021; Brandes et al., 2022; Cui et al., 2024a). Transformers generally outperform LSTMs and similar architectures by offering several key advantages. The primary strength of Transformers lies in their multi-headed attention mechanism. This feature allows a self-attention process that effectively captures long-range dependencies in the data, significantly reducing the ‘forgetting’ issue common in LSTMs and enabling the analysis of longer sequences (Shi et al., 2023). Each head in the multi-headed attention mechanism focuses on a different segment of the input text, fostering a richer and more nuanced understanding of long-range interactions (Chen et al., 2023; Shi et al., 2023). Unlike RNNs, where computation is dependent on the previous step, Transformers allow for parallel processing, making them much more efficient for training, deployment, and scaling up (Chen et al., 2023). Additionally, the self-attention mechanisms within Transformers can be examined to identify which parts of the sequence the model emphasizes, providing insights into the statistical relationships between sequence elements (Choi and Lee, 2023). However, despite these advantages, the attention mechanism’s quadratic complexity in Transformers means that as the sequence length increases, the memory and computational requirements grow quadratically (Shi et al., 2023). This makes Transformers computationally demanding and limits the length of sequences they can feasibly handle.

## What is LLMs?

The use of transformer-based models in biology has led to significant advancements, most notably with the development of AlphaFold2 (AF2) (Jumper et al., 2021), a groundbreaking model for predicting protein structures. While transformers are the core of many language models, they are not universal. For instance, models, such as the DNA LLM HyenaDNA (Nguyen et al., 2024), do not use transformers, and not all transformer-based models qualify as LLMs, with AF2 being a prime example. Although there is no widely accepted threshold distinguishing a standard LM from LLMs, LLMs are generally recognized by their high number of parameters, often in the billions, and are typically trained on large datasets, offering more capabilities than typical LMs (Lam et al., 2024).

LLMs can be broadly categorized into three architectural types: encoder-decoder, encoder-only, and decoder-only models, each tailored for specific applications and strengths (Raiaan et al., 2024). Encoder-decoder models, such as the original Transformer model introduced by Vaswani et al. (2018), excel in tasks that require transforming input data into a desired output format, such as machine translation. These models use an encoder to process and condense input data into an abstract form, which the decoder then uses to produce the output, effectively managing context and relationships within and across sequences. Encoder-only models, such as Bidirectional Encoder Representations from Transformers (BERT) (Kenton and Toutanova, 2019), are optimized for understanding and analyzing information within a sequence, making them ideal for tasks like classification, named entity recognition (NER) (Radford et al., 2018), and summarization. BERT’s architecture captures bidirectional context, allowing it to understand the connections and nuances of words in a text, leading to more accurate interpretations and classifications (Kenton and Toutanova, 2019). Decoder-only models, exemplified by Generative Pre-trained Transformer (GPT) models, excel in generating coherent and contextually relevant text (Barbhuiya et al., 2024). They are primarily used in applications involving text generation and translation, focusing on creating smooth and contextually appropriate content. GPT models use their autoregressive capabilities to predict the next token in a sequence, generating sentences and paragraphs that mimic human writing (Barbhuiya et al., 2024).

Despite their specific designs, these models are flexible and adaptable beyond their original applications. For instance, a fine-tuned version of GPT, such as ChatGPT, can be repurposed for tasks like text classification and NER, often with a high level of accuracy (Raiaan et al., 2024). This is achieved through techniques like zero-shot or few-shot prompting, enabling the model to apply its learned language understanding to new tasks with minimal additional training (Barbhuiya et al., 2024; Raiaan et al., 2024). This adaptability highlights the potential of LLMs to address a variety of challenges across different domains, making them invaluable tools in natural language processing and beyond.

The versatility of LLMs in processing word sequences applies to various types of sequential biological data (Sarumi and Heider, 2024). Both BERT and GPT models have been adapted for genomic, proteomic, and gene expression analyses (Rehana et al., 2023, 2024; Sarumi and Heider, 2024). Typically, LLMs undergo pretraining using self-supervised methods, taking advantage of the wealth of publicly available genomic data. For instance, BERT models use masked language modeling (MLM), predicting masked tokens within a sequence (Rehana et al., 2023). In contrast, GPT models employ causal language modeling, predicting subsequent tokens in a sequence (Sarumi and Heider, 2024). This approach gives GPT its autoregressive capability, as it predicts new words iteratively and incorporates them back into the model to continue generating sequences. Through this pretraining process, LLMs learn intrinsic patterns in the data, which can then be used to extract features and identify patterns in new, unseen data (Sarumi and Heider, 2024). These pretrained foundational models can be further adapted for specific tasks through fine-tuning with supervised learning techniques, expanding their application scope across various domains in biological research.

### Current status of LLMs in biological science

LLMs are making significant strides in the realm of biological sciences, thanks to their sophisticated natural language processing capabilities (Lam et al., 2024). Initially designed to understand and generate human-like text, LLMs have been repurposed to interpret complex scientific literature, providing a valuable asset for researchers (Raiaan et al., 2024). In biology, they are increasingly used for mining scientific texts, extracting relevant knowledge, and identifying patterns across vast corpuses of data (Lam et al., 2024). Their ability to process and synthesize information from disparate sources enables researchers to stay abreast of the latest findings, formulate research questions, and hypothesize based on existing literature (Chen et al., 2021; Lam et al., 2024).

Some of the prominent applications of LLMs in biological sciences include assisting in the annotation of genomic datasets, predicting protein functions, and integrating diverse types of scientific data such as chemical, genetic, and phenotypic information (Lam et al., 2024; Sunil et al., 2024). By facilitating the interpretation of complex biological narratives and enhancing communication between different types of data, LLMs contribute to a more holistic understanding of biological processes. Moreover, their predictive capabilities can be used to predict developments in fields such as drug discovery and personalized medicine, offering potential solutions to pressing health and environmental challenges (Sunil et al., 2024).

Despite their promising applications, the integration of LLMs in biological science is still in its growing stage. Challenges such as data privacy, interpretation accuracy, and the need for domain-specific training data remain (Kuska et al., 2024). Nevertheless, ongoing improvements and adaptations to the unique requirements of biological research are expected to overcome these hurdles. As the scope of LLM applications continues to expand, they are poised to become indispensable tools in the toolkit of biologists, facilitating new discoveries and advancing the field.

### Why LLMs are the future of plant breeding?

As plant breeding evolves into a highly data-driven and precision-oriented domain, the potential of LLMs to fundamentally reshape this field is immense. By offering an unprecedented ability to learn from textual data, LLMs have the capacity to revolutionize how breeders’ access, interpret, and utilize scientific knowledge. These models can serve as intelligent agents capable of integrating and synthesizing vast amounts of historical breeding records, genomic data, and recent scientific publications to inform breeding decisions. The strategic use of LLMs could thus streamline processes like literature reviews, hypothesis generation, and the development of new breeding strategies.

LLMs can significantly augment breeders’ ability to predict outcomes and identify genetic traits associated with yield, stress tolerance, and other important agronomic characteristics. They can analyze and correlate data from large genomic repositories, helping breeders to pinpoint potential genetic markers for selection. Furthermore, as LLMs become more specialized, they could play a crucial role in automating routine tasks such as phenotyping, creating multilingual databases of breeding information, and assisting in cross-disciplinary research by translating domain-specific terminology across scientific fields.

Moreover, the adaptive learning nature of LLMs means they can improve continually as more data becomes available, offering solutions that grow in accuracy and utility over time. Their potential to interface with other technologies such as Internet of Things (IoT) devices for real-time data collection, and CRISPR for precision gene editing, suggests a future where breeders can make faster, more informed decisions that lead to rapid advancements in crop development (Kuska et al., 2024). In this context, LLMs will not only symbolize the future of plant breeding but also act as catalysts for innovations that meet the global agricultural demands of tomorrow.

### Leveraging LLMs and biological language models in plant breeding

Natural language models (NLMs), initially created for understanding and generating human language, are able to transform plant breeding by streamlining access to extensive textual datasets such as research papers, databases, and reports that are publishing every day. It would be challenging for plant breeders to keep up with the pace of publications, therefore, utilizing NLMs would be the best approach to ensure the new information can be consider in the breeding pipeline. These datasets can enhance the understanding of genotype and phenotype of interests, which are fundamental to plant breeding (Busta et al., 2024). Additionally, NLMs can integrate data from genetic markers, phenotypic images, gene sequences, and environmental data, forming multimodal models that deliver more comprehensive insights into crop traits and breeding strategies (Ji et al., 2023). While general NLMs such as GPT and BERT are pre-trained on broad datasets and prove adaptable across various domains, their lack of specialization may result in inaccurate interpretations, particularly in specialized fields including plant breeding (Ji et al., 2023). Specialist NLMs, tailored to specific domains, can be fine-tuned on breeding-related corpora and incorporate key insights about genetic trait correlations and region-specific crop requirements (Rehana et al., 2023).

Beyond text knowledge, NLMs simplify bioinformatics workflows, assisting in coding, debugging, and navigating complex software tools specific to genome-wide studies in plant breeding (Zhao et al., 2023). Similarly, biological language models, trained to process DNA, RNA, or protein sequences, apply principles such as context recognition to predict genetic mutations’ effects on phenotypes and explore gene networks responsible for trait regulation (Lam et al., 2024). These models hold the potential to elevate precision breeding techniques by facilitating cross-species comparisons and identifying conserved traits (Zhao et al., 2023). In this case, text-based embeddings can seamlessly combine with other modalities, such as gene expressions, to enhance candidate gene identification for desirable traits, potentially leading to the development of climate-resilient cultivars (Zhao et al., 2023).

As another area to explore in plant breeding, knowledge graphs, representing entities and relationships as nodes and edges, provide an integrated framework to connect disparate data sources, which is important for linking genetics and environmental parameters in plant breeding. In scientific research, LLMs such as SciBERT, BioBERT, and BioGPT have significantly impacted knowledge graph construction by efficiently extracting entities and their relationships from unstructured text, forming triple-based structured data representations (Bi et al., 2024). The integration of these models with knowledge graphs reduces inaccurate responses and leverages robust reasoning to improve performance in domains requiring precise information retrieval, demonstrating significant advancements in artificial intelligence applications (Lim et al., 2024). Therefore, combining language models with knowledge graphs, particularly through techniques like Think-on-Graph (ToG), allows sophisticated reasoning by extracting multi-hop connections for comprehensive query responses (Sun et al., 2023).

**Figure 3:**
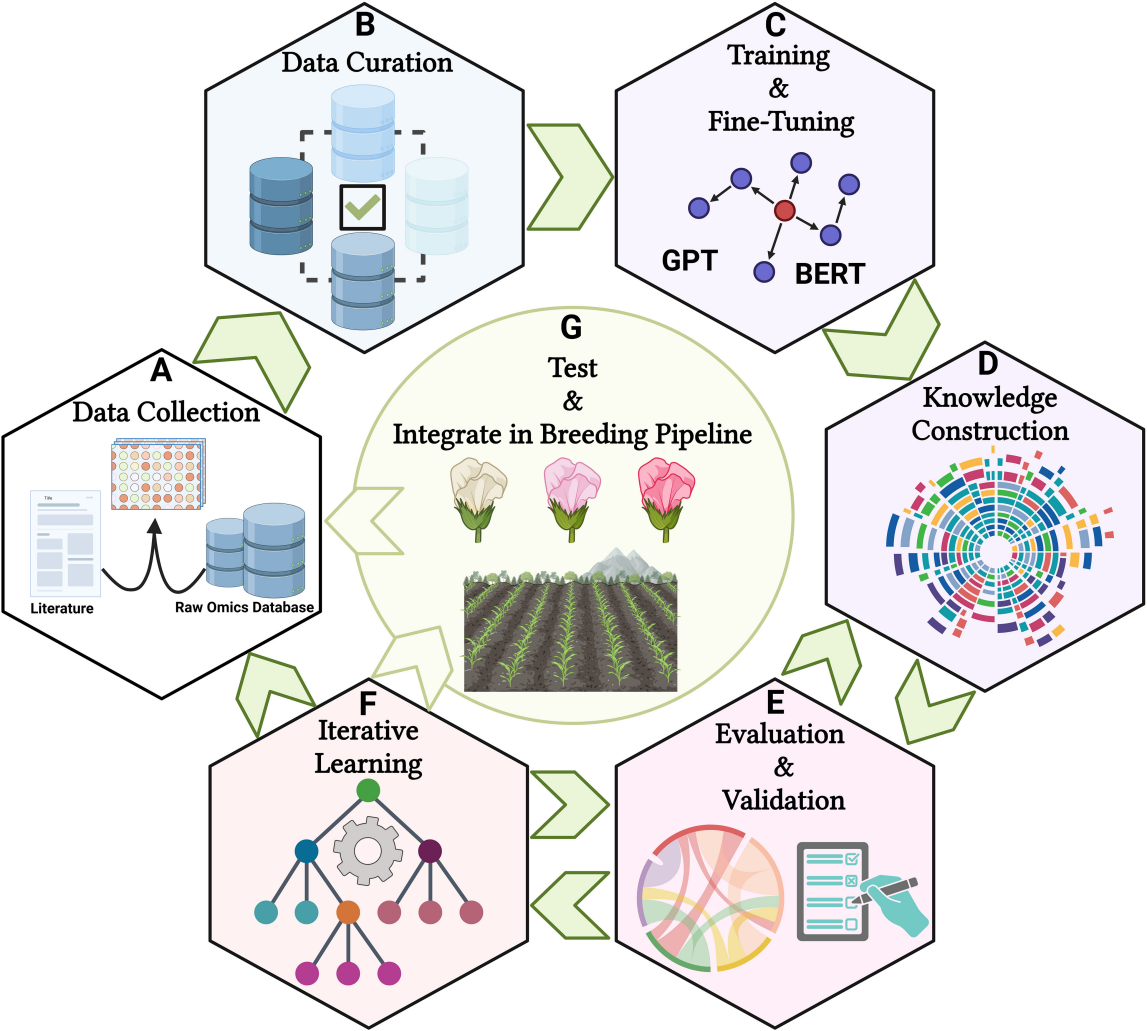
A schematic picture of utilizing LLMs in plant breeding area. **(A)** Collecting diverse datasets from scientific literature, multi-omics repositories, and breeding records, **(B)** Standardizing text and annotating multi-omics data during preprocessing, **(C)** Choosing a pre-trained LLM, fine-tuning it with plant breeding-specific texts, and using multi-modal methods to integrate text and structured data, **(D)** Leveraging the LLM to build knowledge graphs that illustrate the relationships between multi-omics, traits, and environmental factors, **(E)** Establishing performance metrics and refining outputs with input from breeders and biologists, **(F)** Creating feedback loops to continuously assess results, and **(G)** Assisting plant breeders in developing data-driven strategies by prioritizing multi-omics and traits for field trials based on their yield, quality, and adaptability. The figure was created using BioRender.com.

In practice, integrating LLMs in plant breeding necessitates a structured methodology encompassing data collection, model training, and evaluation (Rehana et al., 2024). It begins with curating a comprehensive dataset from literature and multi-omics databases, proceeding with fine-tuning pre-trained LLMs on plant-specific corpora to enhance language understanding and data integration capabilities (**Figure 3**). This seamless integration facilitates hypothesis generation and decision-making, allowing breeders to query the models for insights and strategies that influence breeding multivariate decisions (**Figure 3**). Ultimately, deployment with user training embeds these tools in practical breeding contexts, ensuring their utility through intuitive interfaces and insightful visualizations. Through these concerted operations, language models emerge as valuable assets in precision plant breeding, advancing genetic understanding and breeding innovations (**Figure 3**).

### How to make it feasible to reach crops from codes?

Bridging the gap between advanced computational codes and tangible improvements in crops necessitates a multifaceted approach (S.S et al., 2024). Firstly, an integrated infrastructure that supports data acquisition, storage, processing, and analysis is essential. This calls for investment in robust high-performance computing facilities and cloud-based platforms that can accommodate large-scale datasets and computational processes required for training LLMs and running predictive algorithms (Chen et al., 2024). These infrastructures should be designed to ensure data security, user accessibility, and interoperability across global breeding programs.

Secondly, interdisciplinary collaboration between data scientists, agronomists, geneticists, and breeders is critical for translating computational insights into actionable breeding strategies. Developing user-friendly interfaces and visualization tools can facilitate this collaboration, enabling breeders to interact intuitively with complex data outputs and derive practical insights for field implementation. Training programs and workshops aimed at enhancing the computational literacy of breeders would further enable a seamless transition from theoretical codes to real-world applications.

Moreover, the development of standardized protocols and validation frameworks is vital to ensure the reliability and reproducibility of LLM-driven predictions. Establishing rigorous benchmarks and workflows for model evaluation helps in optimizing the performance and applicability of these systems to diverse crop species and environments. Continuous feedback loops where insights from field trials are used to refine models can enhance the accuracy and relevance of predictions, thus ensuring that computational innovations translate into meaningful crop improvements.

Lastly, fostering a culture of openness and data sharing within the global plant breeding community can accelerate the adoption and optimization of LLM technologies. By sharing successful case studies, datasets, and coding methodologies, stakeholders can collectively advance the state-of-the-art and expedite the realization of LLM-driven breakthroughs in crop science. This collaborative approach not only expedites innovation but also democratizes access to cutting-edge technologies, ensuring that the benefits of research are shared widely across borders and communities.

### How to utilize existing LLM tools in plant breeding area?

Several tools have been recently developed in plant science through the use of LLMs that can be potentially use in plant breeding. PlantConnectome, as an example, utilizes the power of GPT to distill great understanding from approximately 71,000 plant literature abstracts. By constructing a detailed knowledge graph, PlantConnectome has a significant potential to show previously unreported relationships that existing databases have overlooked (Lim et al., 2024). This ability to uncover novel connections can direct breeding programs towards previously unidentified genetic traits that could enhance resistance to diseases or adaptivity to changing climates, proving invaluable in developing new plant varieties with desirable characteristics. Similarly, AgroLD integrates around 900 million triples from over 100 datasets, which synthesizes complementary information for hypothesis formulation and validation (Larmande and Todorov, 2021). In plant breeding area, AgroLD offers breeders a comprehensive resource for identifying genetic markers associated with these traits, thus facilitating targeted breeding strategies for robust crop varieties.

Plant Reactome, as another example, serves as an expansive knowledgebase of plant pathways, offering curated pathways from rice and projections to 129 other species. Its repository of 339 reference pathways provides a detailed view of metabolic processes, hormone signaling, and genetic regulation (Gupta et al., 2024). By facilitating the visualization and analysis of multi-omics data within plant pathways, this resource allows breeders to identify genetic interactions and pathways critical for desired traits such as enhanced yield or stress tolerance, directing breeding efforts more effectively. As another example, WGIE specializes in extracting important wheat germplasm information from fragmented research data (Wei and Fan, 2024). By employing conversational LLMs and innovative data extraction methodologies, WGIE enhances the accessibility and efficiency of identifying useful wheat traits. Such advancements support breeders in selecting the best traits for superior yield and adaptability, addressing both current and future food production demands.

AgroNT pushes the boundary of high-throughput analysis in plant genomics, focusing on crop varieties. It excels in predicting regulatory annotations, promoter strengths, and tissue-specific gene expression, whilst also prioritizing functional variants important for plant breeding (Mendoza-Revilla et al., 2024). Its large-scale application in evaluating mutations can support breeders in selecting beneficial genetic modifications or variants to enhance crop performance under diverse environmental conditions, making it a formidable tool for future agricultural innovations. FloraBERT demonstrates the potential of deep learning models in predicting gene expression by utilizing transfer learning from a wide array of plant species (Levy et al., 2022). This approach surpasses traditional models by providing insights into taxonomic relationships and nucleotide positions within gene promoters. Such insights can be instrumental in guiding plant breeders towards genomic loci that control important phenotypic traits, thereby enhancing the efficiency of breeding programs targeting specific traits.

In general, LLM-based research tools are emerging as powerful resources in plant science, with significant potential to revolutionize plant breeding despite their application in this domain being relatively new and underexplored. Tools such as PlantConnectome, AgroLD, Plant Reactome, WGIE, AgroNT, and FloraBERT have been developed primarily for plant genomics and related fields, with limited direct adoption by plant breeders to date. However, in plant breeding, an LLM could integrate decades of breeding trial data with genomic and phenotypic records to pinpoint genetic markers associated with high yield under various environmental conditions. Similarly, it could analyze unstructured field notes alongside structured datasets to identify management practices, such as optimal planting density or nutrient application, that enhance trait expression across different genotypes. Another possibility is using LLMs to predict phenotypic outcomes by combining historical trial data with current environmental inputs, enabling breeders to prioritize crosses likely to produce resilient lines. Breeders can interact with these models conversationally, posing questions such as, “What genetic factors most influence yield stability in maize?” and receive synthesized responses drawn from diverse data sources, streamlining decision-making and enhancing crop improvement strategies. These applications leverage LLMs’ ability to handle multimodal data and uncover subtle correlations, making them valuable for accelerating breeding cycles and improving selection accuracy without requiring extensive manual preprocessing or specialized computational expertise.

Beyond these general uses, LLMs hold particular promise for deepening the understanding of environmental effects (E) and genotype-by-environment interactions (G×E), which are an integral part of the breeding process. Environmental factors significantly shape phenotypic expression, but their variability and interdependence make them challenging to incorporate into breeding decisions. LLMs can help by processing large-scale environmental datasets alongside genetic and phenotypic data to model G×E interactions with greater precision. For instance, an LLM could integrate historical climate records, soil sensor data, and multi-site trial results to forecast how different genotypes might perform under projected climate change scenarios, aiding breeders in selecting lines with robust adaptability. Additionally, LLMs can analyze diverse data sources, such as satellite imagery or grower observations, to detect environmental patterns linked to desirable traits, such as stress tolerance or nutrient efficiency, and suggest tailored management strategies (M) such as irrigation timing or fertilizer use. By incorporating real-time inputs from IoT devices in fields or greenhouses, LLMs could also provide dynamic recommendations for adjusting breeding trials or phenotyping protocols to account for current conditions. Although their use in these areas is still in its infancy, LLMs’ capacity to manage complex, multimodal data and identify non-linear relationships positions them as a transformative tool for breeders aiming to enhance crop resilience and productivity in the face of environmental uncertainty.

These advancements are not without limitations. The effectiveness of LLMs largely depend on the quality and coverage of the available training datasets. The model predictions in breeding decisions can be biased due to incomplete data, limiting the potential value of the model. Additionally, the computational resources required for training large models can pose accessibility challenges, particularly in regions with limited technological infrastructure. However, there are several ways to effectively measure LLMs into plant breeding workflow. The plant breeding community can enhance the evaluation and maximization of the impact of LLMs, by measuring their performances through objective metrics such as precision, recall and accuracy and by measuring against real world datasets. This not only helps verify that LLMs can provide practical benefits over existing approaches, but also provides insights for how best to improve their use in future applications, thereby further grounding them as drivers of innovation in plant breeding.

### How practical is to build LLMs from scratch?

The emergence and evolution of LLMs over recent years have significantly advanced artificial intelligence capabilities, enabling machines to perform complex language processing tasks with great skill and accuracy (Lam et al., 2024). However, the process of developing an LLM from the scratch involves substantial financial and computational investments. Training these models would be highly expensive, depends on the number of tokens, model’s size and complexity.

In the context of LLMs, a token often represents a unit of text, which can range from a single character to an entire word, depending on the tokenization strategy employed by the model (Yang, 2024). This tokenization process allows models to manage extensive vocabularies while maintaining a relatively fixed size for processing (Men et al., 2024). For example, in OpenAI’s GPT series, text is typically tokenized into subword components, enabling the model to comprehend and generate language with a high degree of flexibility (Bhattacharya et al., 2024). Understanding the role of tokens is crucial because they influence the volume of training data required and, consequently, the model’s overall performance. Calculating the cost of training an LLM primarily involves several factors: the number of tokens, the computational requirements, and the decision of whether to rent or purchase the necessary hardware (Tuggener et al., 2024). Larger training datasets, with their vast token counts, directly impact the amount of computational power needed. For instance, a model with 10 billion parameters might require around 100,000 GPU hours, while a 100 billion parameter model could need as much as one million GPU hours to train. Renting GPUs, such as the high-performance NVIDIA A100, can cost between $1 USD and $2 USD per GPU hour, which translates to training expenses of about $150,000 USD to $1.5 million USD, depending on the model size (Theodoris et al., 2023). Alternatively, purchasing a GPU cluster, potentially consisting of 1,000 GPUs, involves significant upfront costs, estimated at around $10 million USD, excluding operational expenses like energy use. Energy consumption is another critical factor as training large models can consume approximately 1,000 megawatt-hours of energy, adding about $100,000 USD to the expenses at an assumed rate of $100 USD per megawatt-hour. Training large models such as Evolutionary Scale Modeling (ESM-2), a pretrained language models for proteins (Lin et al., 2023), may exceed $200,000 USD. However, pretraining smaller models such as DNABERT-2 (Zhou et al., 2023) or GeneFormer (Cui et al., 2024c) via cloud services can cost several hundred dollars. These considerations help plant breeders assess the financial and logistical requirements of LLM training, directing decisions between developing models in-house or utilizing pre-trained models. Furthermore, the open-source nature of many models comes with comprehensive user guides, simplifying the process of fine-tuning and deployment, especially for computational plant breeders experienced with Python.

Beyond computational demands, the specificity and variability within plant breeding datasets presents challenges for the deployment of LLMs in plant breeding. In this area, the datasets are heterogeneous as they collected from different environments over multiple years, leading to accuracy concerns if LLMs are trained on incomplete or biased data. In order to make sure about the robustness of LLMs, training datasets should be representative, encompassing full genetic diversity as well as comprehensive and broad phenotypic and multi-omics data. Additionally, interpretability is still a problem because it can be challenging to comprehend how LLMs make predictions. Therefore, enhancing interpretability with visualization tools and explainable AI methods is vital for making LLMs more accessible and actionable for plant breeders. Additionally, integrating LLMs into existing processes requires careful consideration of data privacy and ethical implications to preserve breeder autonomy and respect original knowledge. Addressing these challenges will help optimize LLM benefits while mitigating their limitations in plant breeding.

Despite the high resource demands and complexity associated with creating a LLM for plant breeding, the potential benefits are profound. A model trained on vast range of datasets from plant breeding and multi-omics could significantly enhance scientific research, significantly speed up the crop improvement by making breeder’s decision more accurate. This potential is exemplified in the ongoing expansion of plant LLMs, such as FloraBERT (Levy et al., 2022) and AgroNT (Mendoza-Revilla et al., 2024). Yet, current efforts predominantly focus on model creation, with less emphasis on training with in-depth plant data and even fewer on practical applications in plant research. The sequencing of over 788 plant genomes presents a vast opportunity for pretraining models across a wide variety of plant groups (Cui et al., 2024b). Moreover, with the increasing availability of single-cell RNA-sequencing data, these models can be pretrained and fine-tuned with additional modalities, such as those capturing the epigenome, proteome, and metabolome. As an example, *Arabidopsis thaliana* alone boasts over one million sequenced nuclei, supporting extensive research in plant development and responses to environmental factors (Nobori et al., 2023). Studies have produced comprehensive atlases encapsulating seed-to-seed development and various responses in root systems and leaves (Lee et al., 2023; Nobori et al., 2023). These growing datasets present an invaluable resource for the progressive training and refinement of plant breeding LLM.

An important factor in the effectiveness of any LLM is the quality and breadth of its training data (Feng et al., 2023). The concept of “garbage in, garbage out” is particularly relevant, indicating that the output quality directly reflects the input’s quality. To create an LLM beneficial for plant breeding, access to diverse and rich datasets is vital (Farooq et al., 2024a). These should include genetic sequences, phenotype information, climate data, and a broad spectrum of scientific literature. Although platforms like NCBI, Common Crawl (Patel and Patel, 2020) or commercially available datasets such as C4 provide a starting point, it is imperative to ensure data integrity and relevance. Additionally, adherence to legal standards, particularly concerning copyright regulations, is a necessary consideration in data collection and usage.

Optimizing LLM training involves leveraging sophisticated techniques that streamline processes and minimize costs (Shahini et al., 2024). One such method is mixed precision training, which combines 16-bit and 32-bit floating-point numbers to manage computational demands efficiently (Parthasarathy et al., 2024; Shahini et al., 2024). This approach, along with 3D parallelism strategies, enables the creation of robust, scalable models equipped to handle extensive datasets typical in plant breeding. Upon training an LLM, thorough evaluation is essential to determine its efficacy for targeted applications in plant breeding. Performance benchmarks tailored to areas such as plant breeding or bioinformatics can be instrumental in assessing the model’s accuracy and adaptability. Following the evaluation phase, fine-tuning the model through techniques such as prompt engineering or targeted adjustments allows it to home in on specific tasks, whether predicting plant traits based on genetic data or integrating recent findings from breeding studies.

## Conclusion

The evolution of LLMs can revolutionize plant breeding by providing breeders with new tools for discovering and incorporating large and diverse amounts of data. To efficiently utilize LLMs in plant breeding programs, plant breeders should identify specific areas in their breeding objectives where LLMs can add value, such as uncovering new genetic interactions or improving predictions of traits. The next step is effective data preparation, including curating high-quality, diverse datasets that accurately represent genetic variation and environmental factors. Best practices include working with data scientists to improve the quality of the data and using publicly available multi-omics databases to pre-train LLMs. Overall, LLMs have the potential to benefit breeders through enhanced predictive accuracy and automation of data analysis, which reduces dependence on trial and error. Through the application of visualization tools and explainable AI methods, LLM outputs will be significantly interpretable, facilitating informed decisions. Ongoing model validation with real data will also ensure pragmatic applicability and effectiveness. As these technologies evolve, engaging with LLM-driven research communities will foster shared learning and innovation. By implementing these steps, plant breeders can better integrate LLMs within their programs, paving the way for a data-driven, precision breeding era. These advancements contribute to sustainable agriculture and global food security, making the breeding process more dynamic and responsive to future needs.
